# Epstein-Barr virus (EBV) activates NKL homeobox gene HLX in DLBCL

**DOI:** 10.1371/journal.pone.0216898

**Published:** 2019-05-29

**Authors:** Stefan Nagel, Cord C. Uphoff, Wilhelm G. Dirks, Claudia Pommerenke, Corinna Meyer, Hans G. Drexler

**Affiliations:** Department of Human and Animal Cell Lines, Leibniz-Institute DSMZ – German Collection of Microorganisms and Cell Cultures, Braunschweig, Germany; University of North Carolina at Chapel Hill, UNITED STATES

## Abstract

NKL homeobox genes encode developmental transcription factors regulating basic processes in cell differentiation. According to their physiological expression pattern in early hematopoiesis and lymphopoiesis, particular members of this homeobox gene subclass constitute an NKL-code. B-cell specific NKL-code genes generate a regulatory network and their deregulation is implicated in B-cell lymphomagenesis. Epstein-Barr virus (EBV) infects B-cells and influences the activity of signalling pathways including JAK/STAT and several genes encoding developmental regulators. Therefore, EBV-infection impacts the pathogenesis and the outcome of B-cell malignancies including Hodgkin lymphoma and diffuse large B-cell lymphoma (DLBCL). Here, we isolated EBV-positive and EBV-negative subclones from the DLBCL derived cell line DOHH-2. These subclones served as models to investigate the role of EBV in deregulation of the B-cell specific NKL-code members HHEX, HLX, MSX1 and NKX6-3. We showed that the EBV-encoded factors LMP1 and LMP2A activated the expression of HLX via STAT3. HLX in turn repressed NKX6-3, SPIB and IL4R which normally mediate plasma cell differentiation. In addition, HLX repressed the pro-apoptotic factor BCL2L11/BIM and hence supported cell survival. Thus, EBV aberrantly activated HLX in DLBCL, thereby disturbing both B-cell differentiation and apoptosis. The results of our study appreciate the pathogenic role of EBV in NKL homeobox gene deregulation and B-cell malignancies.

## Introduction

Hematopoietic stem cells reside in the bone marrow and generate precursor cells for the myeloid and lymphoid lineages. The last steps of B-cell development take place in the germinal centers which are located in lymphoid organs. They include the differentiation into plasma cells (CD38+ CD138+ surface IgG-) or memory B-cells (CD38- CD138- surface IgG+) which express particular cell type specific factors. These developmental processes are mainly regulated at the transcriptional level. Accordingly, several transcription factors like BCL6 and PAX5 act as master genes/factors for B-cell development [1,2]. Moreover, their deregulation or mutation contributes to cell transformation and lymphomagenesis [3]. Recently, we have described four members of the NKL homeobox gene subclass which are expressed in the course of B-cell development [4]. These B-cell associated genes display together with additional NKL homeobox genes expressed in early hematopoiesis and T-cell lymphopoiesis a specific pattern that we have termed NKL-code [4,5]. Deregulation of these nine code-members or aberrant activation of non-hematopoietic NKL homeobox genes seems to be involved in the generation of leukemia and lymphoma [4,5]. Prominent examples for B-cell malignancies that aberrantly overexpress NKL-code members HLX and NKX2-3 are Hodgkin lymphoma (HL) and splenic marginal zone lymphoma [6,7]. Furthermore, subsets of diffuse large B-cell lymphoma (DLBCL) and HL ectopically express the non-code members NKX2-1 and NKX2-2, respectively [8,9].

DLBCL is the most common type of B-cell malignancies [10]. This disease has been categorized into different subtypes according to expression profiling data, IRF4-rearrangement, translocations targeting MYC, BCL2 and/or BCL6, and Epstein-Barr virus (EBV) infection [10]. Thus, clinical manifestations of DLBCL are associated with several factors which influence the prognosis and the survival of the patients.

EBV is a 172 kb long DNA-virus that belongs to the group of human herpesviruses and is accordingly also named HHV4. It encodes more than 80 genes and enters epithelial and lymphoid cells via the complement receptor CR2/CD21 [11–13]. Infections of B-cells with EBV are widespread and the course of the provoked disease is mostly asymptomatic. Nevertheless, this virus is associated with several B-cell malignancies including Burkitt lymphoma, HL, and DLBCL [14]. Important EBV-encoded proteins in this context are EBER2, EBNA1, EBNA2, EBNA3C, LMP1 and LMP2A. They have been shown to deregulate developmental genes which play fundamental roles in B-cell differentiation including BACH2, BCL6, IRF4, PAX5, PRDM1 and STAT3 [15–20]. EBV can exhibit one of three latency programs which differ in expression of particular EBV-encoded genes. EBNA1 is expressed in all three latency programs, EBNA2, EBNA3A and EBNA3C are expressed in latency program 3, and LMP1 and LMP2A in latency programs 2 and 3 [11,21]. Furthermore, NFkB- and JAK-STAT-pathways are aberrantly activated by EBV as well [22]. All these alterations are etiologically connected with B-cell lymphomagenesis [3,14]. The presence of EBV in DLBCL is associated with a worse outcome [23], highlighting the demand for novel rational therapeutic options.

Here, we established a cell line model to analyze the EBV-mediated impact on B-cell differentiation factors in DLBCL. We show the mechanism and the consequences of EBV in deregulation of the NKL-code member HLX.

## Materials and methods

### Cell culture and treatments

EBV-negative (ACC No. 47) and EBV-positive subclones of the DLBCL cell line DOHH-2 are held by the DSMZ (Braunschweig, Germany) and were cultivated as described [24]. The testing for authenticity of the DOHH-2 subclones was performed as described previously and confirmed as shown in S1 Fig [25]. The cells were cultured in 24-well plates and photographed using microscope Primovert and associated software ZEN version 2.3 (Zeiss, Göttingen, Germany). Cell stimulations were performed by treatment with 10 μg/ml trichostatin A (TSA, Sigma, Taufkirchen, Germany), with 100 μM AG490 (Sigma), and with 100 μM etoposide (Sigma). Gene specific siRNA oligonucleotides and AllStars negative Control siRNA (siControl) were obtained from Qiagen (Hilden, Germany). The unmodified RNA-oligonucleotide sequences for LMP1 were 5´-GAGACCUUCUCUGUCCACUTT-3´ and 5´-AGUGGACAGAGAAGGUCUCTT-3´, and for LMP2A 5´-CUCCCAAUAUCCAUCUGCUTT-3´ and 5´-AGCAGAUGGAUAUUGGGAGTT-3´ [26]. Expression constructs for LMP2A, EBNA2 and the control vector were obtained from Origene (Wiesbaden, Germany). SiRNAs (80 pmol) and expression constructs/vector controls (2 μg) were transfected into 1x10^6^ cells by electroporation using the EPI-2500 impulse generator (Fischer, Heidelberg, Germany) at 350 V for 10 ms. Transfected cells were harvested after 20 h cultivation. For functional examinations treated cells were analyzed by the IncuCyte S3 Live-Cell Analysis System (Essen Bioscience, Hertfordshire, UK). For detection of apoptotic cells we additionally used the IncuCyte Caspase-3/7 Green Apoptosis Assay diluted at 1:2000 (Essen Bioscience).

### Detection of EBV

The detection of EBV in DOHH-2 cells was performed by interphase fluorescence in situ hybridization (FISH) and by polymerase chain-reaction (PCR) analysis as described recently [27]. Briefly, 2 x 10^5^ DOHH-2 cells were washed with phosphate-buffered-saline buffer (PBS) and centrifuged onto acid washed slides. The cells were fixed with methanol / acetic acid (3:1) and subsequently dehydrated via an ethanol series. Cy3 labeled EBV cosmid DNA and Cot DNA (Roche, Penzberg, Germany) were added to the air-dried slides, denatured and hybridized overnight at 37°C. The slides were then washed with 2xSSC, mounted with Vecta-Shield (Hoechst, Frankfurt, Germany) and evaluated with fluorescence microscope Axiovert 40 CFL (Zeiss) and software VisiView version 1.6.9 (Visitron Systems, Puchheim, Germany).

For PCR analysis, genomic DNA of the cell lines was applied for the detection of episomal, linear, or integrated EBV sequences. The DNA was extracted from PBS washed cells using the High Pure PCR Template Preparation Kit (Roche). The integrity and quality of the DNA was verified by PCR analysis of the human ABL1 gene. The EBV specific PCR primers amplify a 265 bp fragment of a conserved region of the EB2/BMLF1 gene. A parallel reaction was performed with an internal control sequence of 445 bp and amplified with the same primers as the EBV wild type DNA fragment serving as internal control.

Reverse transcription (RT)-PCR was performed using taqpol (Qiagen) and the thermocycler TGradient (Biometra, Göttingen, Germany). The oligonucleotides used for analysis of expressed EBV-encoded genes and a cell line encoded control gene (YY1) were as follows: LMP1-forward 5´-GGAGGCCTTGGTCTACTCCTAC-3´, LMP1-reverse 5´-CGATGAGTAGGAGGGTGACTGG-3´, LMPA2-forward 5´-CCTAGAAATGGTGCCAATGGGC-3´, LMPA2-reverse 5´-GTGTTCCCATAAGAGTCAGAAGC-3´, EBNA1-forward 5´-GATGGTGAGCCTGACGTGCC-3´, EBNA1-reverse 5´-TTGCGCCTGCCTCCATCACC-3´, EBNA2-forward 5´-CGGTTCACCTTCAGGGCCTAGG-3´, EBNA2-reverse 5´-CTGGTAGGACTGGGCGACCGG-3´, EBNA3A-forward 5´-ACCCTGGCCGCCGGATGGCC-3´, EBNA3A-reverse 5´-ACCTGTGCACCCAAGTGTCGCC-3´, EBNA3C-forward 5´-GTGGACACCACCCCATGCTGG-3´, EBNA3C-reverse 5´-GATTCTTCGGTACCGCCTCTGCC-3´, YY1-forward 5´-AAGCAGGTGCAGATCAAGAC-3´, YY1-reverse 5´-CCGAGTTATCCCTGAACATC-3´. The oligonucleotides were obtained from Eurofins MWG (Ebersberg, Germany). The generated PCR products were subsequently analyzed by agarose gel electrophoresis. Documentation was performed using the Azure c200 Gel Imaging System (Azure Biosystems, Dublin, CA, USA).

### Real-time quantitative (RQ) PCR analyses

Total RNA was extracted from cell line samples using TRIzol reagent (Invitrogen, Darmstadt, Germany). Primary human total RNA was commercially obtained. We used RNA from peripheral blood mononuclear cells (PBC), lymph node (LN), spleen (SP), and bone marrow (BM) obtained from Biochain/BioCat (Heidelberg, Germany), and RNA from peripheral CD19-positive B-cells and CD3-positive T-cells obtained from Miltenyi Biotec (Bergisch Gladbach, Germany). cDNA was synthesized from 5 μg RNA by random priming using Superscript II (Invitrogen). RQ-PCR analysis was performed with the 7500 Real-time System, using commercial buffer and primer sets (Thermo Fisher Scientific, Darmstadt, Germany). Quantification of MSX1 was performed as described previously [28]. For normalization of expression levels we analyzed the transcript of TATA box binding protein (TBP). We used the ddCT-method and the obtained values are indicated in relation to one sample which was set to 1. Quantitative analyses were performed in triplicate. Standard deviations are presented in the figures as error bars. Statistical significance was assessed by t-Test and the calculated p-values indicated by asterisks (* p<0.05, ** p<0.01, *** p<0.001, n.s. not significant).

### Protein analyses

Western blots were generated by the semi-dry method. Protein lysates from cell lines were prepared using SIGMAFast protease inhibitor cocktail (Sigma). Proteins were transferred onto nitrocellulose membranes (Bio-Rad, München, Germany) and blocked with 5% dry milk powder dissolved in PBS. The following antibodies were used: alpha-Tubulin (Sigma), BCL2L11/BIM (Thermo Fisher), IL4R (R&D Systems, Wiesbaden, Germany), SMAD1 (Santa Cruz Biotechnology, Heidelberg, Germany), STAT3 (Cell Signaling Technology, Danvers, MA, USA), phospho-STAT3 (Ser727) (Cell Signaling Technology), XBP1 (Santa Cruz Biotechnology). For loading control blots were reversibly stained with Poinceau (Sigma) and detection of alpha-Tubulin (TUBA) was performed thereafter. Secondary antibodies were linked to peroxidase for detection by Western-Lightning-ECL (Perkin Elmer, Waltham, MA, USA). Documentation was performed using the digital system ChemoStar Imager (INTAS, Göttingen, Germany). Densitometric quantification of protein bands was performed using software AzureSpot version 14 (Azure Biosystems). The obtained densities from the protein of interest were correlated to the control bands of TUBA and the calculated values indicated below.

Immuno-cytology was performed as follows: cells were spun onto slides and subsequently air-dried and fixed with methanol/acetic acid for 90 s. The phospho-STAT3 antibody (Cell Signaling Technology) was diluted 1:20 in PBS containing 5% BSA and incubated for 30 min. Washing was performed 3 times with PBS. Preparations were incubated with secondary antibody (diluted 1:100) for 20 min. To detect IgG surface immunoglobulins we used directly labelled anti-IgG antibody diluted at 1:100 (Southern Biotech, Birmingham, AL). After final washing the cells were mounted in Vectashield (Vector Laboratories, Burlingame, CA), containing DAPI for nuclear staining. Documentation of subcellular protein localization was performed using fluorescence microscope Axio-Imager (Zeiss, Göttingen, Germany) configured to a dual Spectral Imaging FISH system (Applied Spectral Imaging, Neckarhausen, Germany).

### Expression profiling

Expression profiling datasets of EBV-positive/negative DOHH-2 subclones using HG U133 Plus 2.0 gene chip (Affymetrix) were generated by Dr. Robert Geffers (Genome Analytics, Helmholtz Centre for Infection Research, Braunschweig, Germany). The primary data are available at Gene Expression Omnibus (www.NCBI.NLM.gov/GEO) via GSE125420. After RMA-background correction and quantile normalization of the spot intensities, the profiling data were expressed as ratios of the sample mean and subsequently log2 transformed. Data processing was performed via R/Bioconductor using limma and affy packages.

For creation of heat maps showing selected values of gene expression profiling data we used the software CLUSTER (version 2.11) and TREEVIEW (version 1.60) originally developed by Michael Eisen (http://bonsai.hgc.jp/~mdehoon/software/cluster/index.html).

Public expression profiling datasets GSE3996 and GSE17372 were exploited for HLX expression (probe set ID: 214438_at) in HL and DLBCL patients, respectively. For statistical calculations and data presentations in box plots we used R-based tools.

## Results

### Characterization of a cell line model for EBV in DLBCL

**Fig 1:**
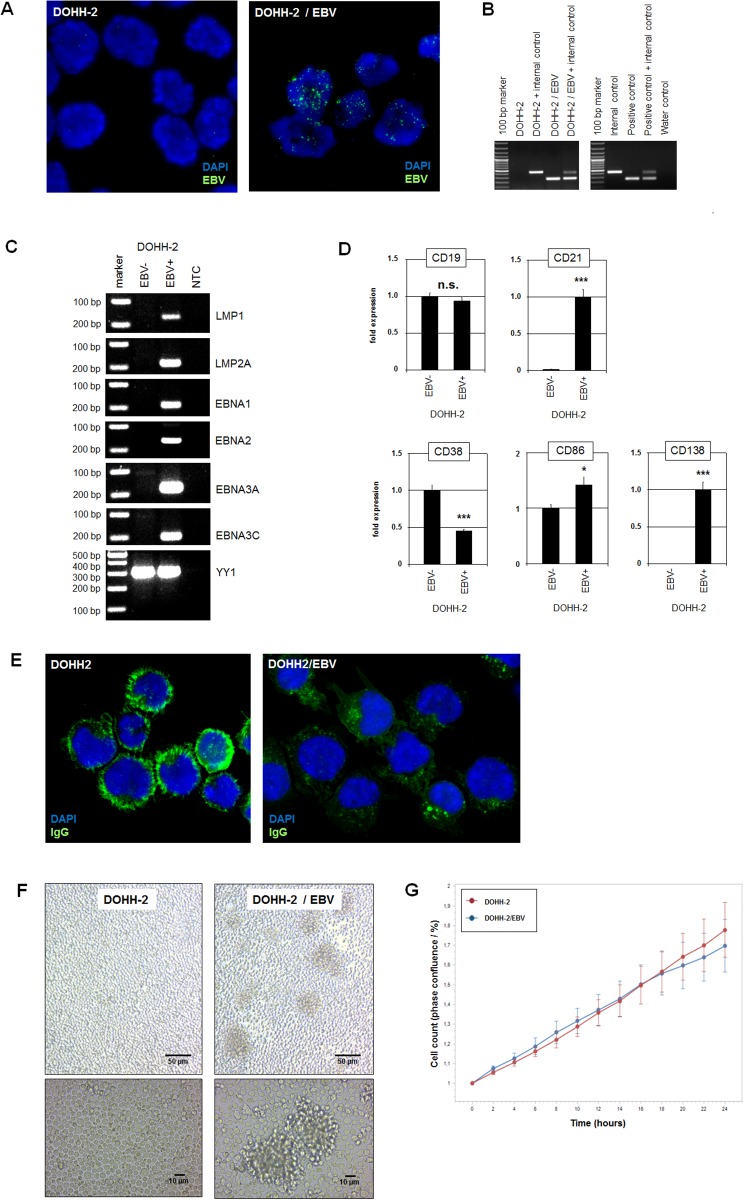
Characterization of EBV-positive/negative DOHH-2 subclones. (A) FISH analysis of EBV in isolated DOHH-2 subclones. The EBV-probe was labelled in green and the nuclei were counterstained with DAPI in blue. (B) PCR analysis of EBV-DNA in isolated subclones. Both procedures confirmed the presence and absence of EBV and thus the purity of the isolated DOHH-2 subclones. (C) RT-PCR analysis of selected EBV-encoded genes showing activity for LMP1, LMP2A, EBNA1, EBNA2, EBNA3A and EBNA3C in EBV-positive DOHH-2 cells. The gene YY1 served as positive control, NTC: no template control. (D) RQ-PCR analysis of CD19, CD21, CD38, CD86 and CD138 in EBV-negative and EBV-positive DOHH-2 subclones. (E) Immuno-fluorescence microscopy analysis was performed for IgG immunoglobulins (green) in EBV-negative (left) and EBV-positive (right) DOHH-2 cells. The nuclei were counterstained by DAPI (blue). (F) Microscopical analysis of living EBV-negative and EBV-positive DOHH-2 subclones in culture. Note that cell aggregates are only visible in EBV-positive DOHH-2 cultures. (G) Quantitative analysis via live-cell imaging analysis of EBV-negative and EBV-positive DOHH-2 subclones indicated no difference in their rates of proliferation.

In a previously published study about the virus load in cell lines we described DLBCL cell line DOHH-2 to contain EBV in less than 5% of the cells, demonstrating that this cell culture consisted of both EBV-positive and EBV-negative cells [27,29]. Here, we performed limited dilution of this cell line to end up with pure EBV-positive and EBV-negative subclones. Subsequent FISH and PCR analyses confirmed the presence and absence of EBV in all cells of the isolated subclones (Fig 1A and 1B). Furthermore, the authentication for both subclones to represent cell line DOHH-2 was demonstrated by STR-profiling (S1 Fig). To determine the latency program operating in EBV-positive DOHH-2 subclones we performed RT-PCR analysis of selected EBV-encoded genes (Fig 1C). The data demonstrated activity of all six analyzed genes including LMP1, LMP2A, EBNA1, EBNA2, EBNA3A and EBNA3C corresponding to latency program 3 [11,21].

EBV particles enter B-cells via the complement receptor CR2/CD21 [13]. Quantification of CD21 expression by RQ-PCR demonstrated high levels in EBV-positive DOHH-2 cells while expression in EBV-negative DOHH-2 cells was barely detectable (Fig 1D). In contrast, the expression levels of the B-cell receptor cofactor CD19 were similar in both DOHH-2 subclones (Fig 1D). Thus, these data support that only CD21-positive B-cells are prone to EBV infection. EBV mediates suppression of plasma cell differentiation [30]. Accordingly, in EBV-positive DOHH-2 cells the expression level of the plasma cell marker CD38 was downregulated and of the memory B-cell marker CD86 upregulated (Fig 1D). However, the plasma cell marker CD138 was increased in EBV-positive DOHH-2 cells (Fig 1D). Furthermore, IgG surface immunoglobulins were expressed much weaker in EBV-positive DOHH-2 cells indicating the phenotype of plasma cells (Fig 1E). Thus, these data show an inconsistent picture of analyzed differentiation markers in both subclones.

EBV-negative DOHH-2 cells grew individualized in suspension while EBV-positive DOHH-2 cells created aggregates (Fig 1E). Similar cell aggregates have been detected in other EBV-positive cell lines as well possibly representing a hallmark of cell cultures infected with this virus [31,32]. However, the proliferation rates of EBV-positive and EBV-negative DOHH-2 cells showed no significant difference as analyzed by quantitative in vivo imaging (Fig 1F). Thus, we isolated two subclones of DLBCL-derived cell line DOHH-2 distinguished by the presence/absence of EBV, a modified morphology of the cell culture, and by gene activities related to plasma cells or memory B-cells. This cell line model was subsequently used to investigate the role of EBV in the regulation of developmental genes in malignant B-cells.

### Identification of genes deregulated by EBV

**Fig 2:**
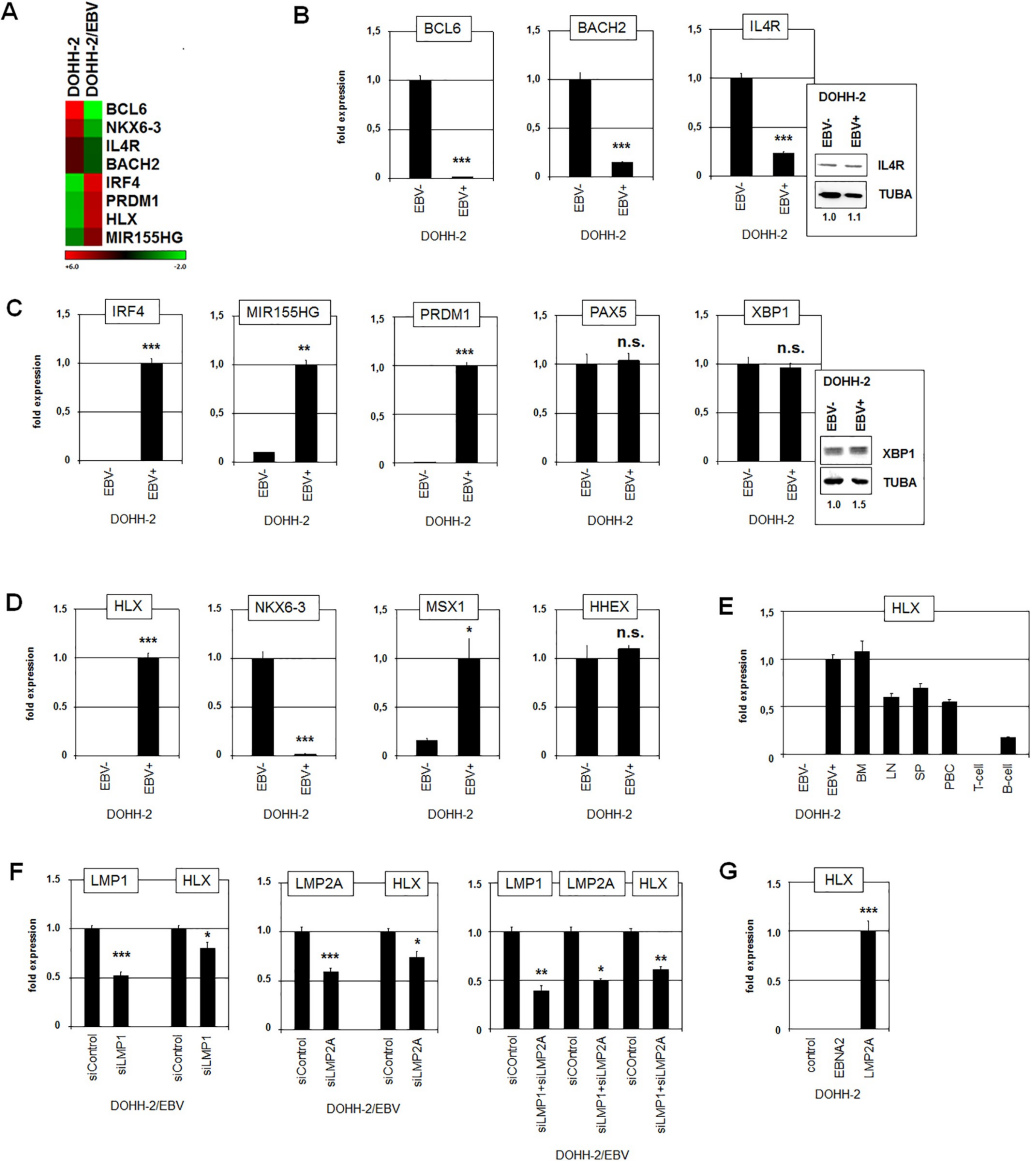
Gene expression analyses in EBV-positive/negative DOHH-2 subclones. (A) The heat map was generated from expression profiling data and shows the expression levels of selected developmental genes in EBV-positive and EBV-negative DOHH-2 subclones. (B) RQ-PCR analysis of BCL6, BACH2 and IL4R demonstrated elevated expression levels in EBV-negative DOHH-2 cells. Western blot analysis was additionally performed for IL4R in EBV-negative and EBV-positive DOHH-2 cells. Tubulin alpha (TUBA) served as loading control, densitometric calculations are indicated below. (C) RQ-PCR analysis of IRF4 and MIR155HG demonstrated elevated expression levels in EBV-positive DOHH-2 cells. Western blot analysis was additionally performed for XBP1 in EBV-negative and EBV-positive DOHH-2 cells. TUBA served as loading control, densitometric calculations are indicated below. (D) RQ-PCR analysis of NKL-code members demonstrated elevated expression levels of HLX and MSX1 in EBV-negative DOHH-2 cells. NKX6-3 expression levels were reduced in EBV-positive DOHH-2 cells while HHEX showed no difference. (E) RQ-PCR analysis of HLX expression in DOHH-2 subclones in comparison to primary hematopoietic cells/tissues including bone marrow (BM), lymph node (LN), spleen (SP), peripheral mononuclear blood cells (PBC), T-cells, and B-cells. (F) RQ-PCR analysis of EBV-positive DOHH-2 cells treated for siRNA-mediated knockdown of EBV-encoded factors LMP1 and LMP2A. The results from a combined knockdown of LMP1 and LMP2A are shown on the right. (G) Forced expression of EBV-encoded EBNA2 and LMP2A in EBV-negative DOHH-2 cells resulted in induced expression of HLX just by LMP2A.

EBV influences the activity of basic B-cell specific genes including BCL6, BACH2, IRF4, MIR155HG and PRDM1 [15,16,18,33,34]. Accordingly, expression profiling data generated from EBV-positive and EBV-negative DOHH-2 subclones showing the top-1000 differentially expressed genes reflected their deregulation (S1 and S2 Tables, Fig 2A). In addition, we confirmed the differing expression levels of these genes by RQ-PCR. The EBV-positive subclone expressed significantly decreased levels of BCL6, BACH2 and IL4R (Fig 2B) while the genes for IRF4, MIR155HG and PRDM1 showed elevated transcript levels (Fig 2C). Western blot analysis of IL4R showed no significant difference at the protein level (Fig 2B), indicating operations of post-transcriptional regulation. PAX5 and XBP1 represent additional factors influenced by EBV [17,35]. However, RQ-PCR and Western blot analyses showed no significant difference between EBV-positive and EBV-negative subclones (Fig 2C). Overall, these data supported the suitability of this cell line model to investigate deregulation of developmental genes caused by EBV.

Recently, we identified a hematopoietic NKL-code which describes the physiological activity of NKL homeobox genes in lymphopoiesis. The B-cell associated code members comprise HHEX, HLX, MSX1 and NKX6-3 and generate a gene network by mutual regulation [4]. RQ-PCR analysis of these genes in EBV-positive/negative DOHH-2 cells demonstrated significant differences for HLX, NKX6-3 and MSX1 while the levels of HHEX were similar in both subclones (Fig 2D). The strongest differences showed HLX and NKX6-3 as supported by the top-1000 expression profiling data (S1 and S2 Tables, Fig 2A). These genes encode mutual repressors in B-cells while MSX1 is suppressed by both HLX and NKX6-3 [4]. As compared to primary hematopoietic cells/tissues, EBV-positive DOHH-2 cells expressed enhanced levels of HLX, resembling those in bone marrow and exceeding the levels in lymph nodes and spleen (Fig 2E). Therefore, our expression data indicated that EBV mediated an activation of HLX which in turn repressed NKX6-3. In addition, reduced NKX6-3 levels might have contributed to elevated MSX1 transcription.

To confirm the role of EBV on HLX we performed siRNA-mediated knockdown of two basic EBV-encoded factors, LMP1 and LMP2A (Fig 2F). The results demonstrated concomitant downregulation of HLX. Combined knockdown of LMP1 and LMP2A further decreased HLX expression supporting the notion that both factors were responsible for enhanced expression of HLX in EBV-positive DOHH-2 cells. Accordingly, transfection of expression constructs for LMP2A (but not for EBNA2) into EBV-negative DOHH-2 cells induced expression of HLX (Fig 2G), confirming the activating impact of this viral factor. Thus, activation of HLX represents a newly discovered consequence of EBV-infection which subsequently deregulates the B-cell associated NKL-code members NKX6-3 and MSX1.

### EBV activates HLX via STAT3

**Fig 3:**
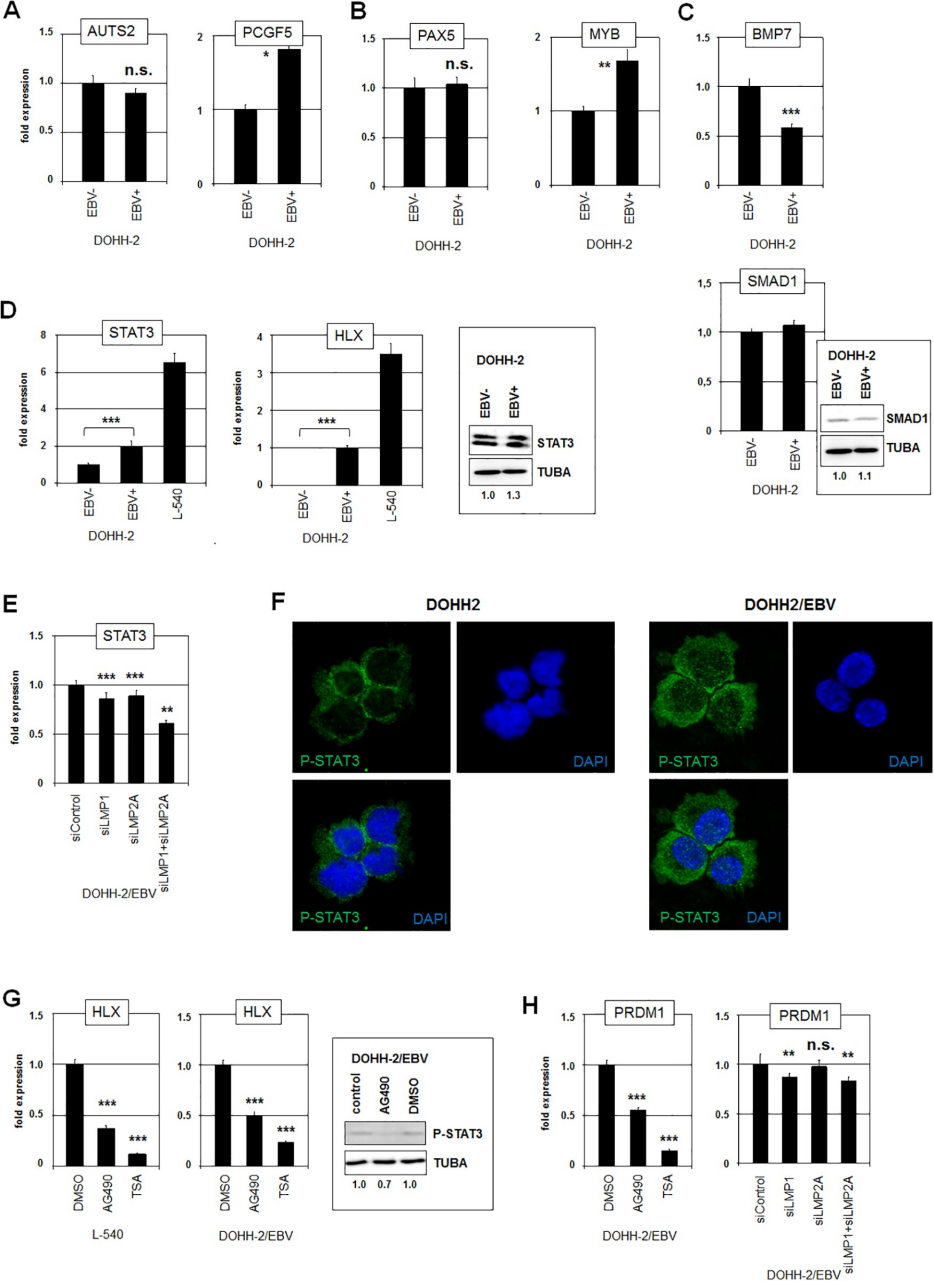
STAT3 activates HLX expression in DOHH-2. RQ-PCR analysis of EBV-negative and EBV-positive DOHH-2 cells for (A) chromatin regulators AUTS2 and PCGF5, (B) transcription factors PAX5 and MYB, and (C) signalling factors BMP7 and SMAD1 (below). Western blot analysis was additionally performed for SMAD1 in EBV-negative and EBV-positive DOHH-2 cells. Tubulin alpha (TUBA) served as loading control, densitometric calculations are indicated below. (D) RQ-PCR analysis of STAT3 (left) and HLX (middle) in EBV-negative and EBV-positive DOHH-2 cells in addition to L-540 cells. Western blot analysis of STAT3 was performed in EBV-negative and EBV-positive DOHH-2 cells (right). TUBA served as loading control, densitometric calculations are indicated below. (E) RQ-PCR analysis of EBV-positive DOHH-2 cells treated for siRNA-mediated knockdown of EBV-encoded factors LMP1 and LMP2A for STAT3. (F) Immuno-fluorescence microscopy analysis was performed for phospho-STAT3 (green) in EBV-negative (left) and EBV-positive (right) DOHH-2 cells. The nuclei were counterstained by DAPI (blue). Note, that the nuclei of EBV-positive cells show elevated levels of phospho-STAT3. (G) RQ-PCR analysis of HLX in L-540 (left) and EBV-positive DOHH-2 cells (middle) treated with STAT3-inhibitors AG490 and TSA for 20 h. Western blot analysis (right) of phospho-STAT3 shows reduced levels in the AG490-treated cells as compared to the controls. TUBA served as loading control, densitometric calculations are indicated below. (H) RQ-PCR analysis of PRDM1 in EBV-positive DOHH-2 cells treated with STAT3-inhibitors AG490 and TSA for 20 h. RQ-PCR analysis of EBV-positive DOHH-2 cells treated for siRNA-mediated knockdown of EBV-encoded factors LMP1 and LMP2A for PRDM1.

Next, we wanted to define the molecular mechanisms of EBV-mediated deregulation of HLX. In addition to the mutual regulation of NKL-code members NKX6-3 and MSX1, we have described several factors which influence the activity of NKX6-3 in B-cells which may thus contribute indirectly to HLX activation [4]. Therefore, we quantified the activities of their genes which encode the chromatin mediators AUTS2 and PCGF5 (Fig 3A), the transcription factors PAX5 and MYB (Fig 3B), and the signalling pathway components BMP7 and SMAD1 (Fig 3C). The RNA-level of the NKX6-3 repressor PCGF5 was higher in EBV-positive cells but that of its activator MYB as well. In contrast, NKX6-3 activator SMAD1 showed no differences at both RNA- and protein-level albeit its activator BMP7 and its repressor MIR155HG were differently expressed [36]. Thus, although some of these genes showed slight differences in their expression levels between EBV-positive and EBV-negative DOHH-2 subclones, these results did not convincingly explain the observed deregulation of NKX6-3 and HLX.

Activation of the JAK/STAT-pathway is a general feature of EBV in DLBCL [22]. In particular, the EBV-encoded factors LMP1 and LMP2A are described to activate the transcription factor STAT3 [37–39]. This fact was of special interest because STAT3 activates the expression of HLX in HL cell line L-540 [6]. Therefore, we speculated if EBV-mediated activation of STAT3 underlies the raise of HLX expression in DOHH-2 cells.

To investigate this hypothesis we first quantified the concomitant expression levels of STAT3 and HLX in EBV-positive and EBV-negative DOHH-2 cells in comparison to HL cell line L-540 (Fig 3D). These data demonstrated a strong positive correlation between the gene activities of STAT3 and HLX. Moreover, EBV-positive DOHH-2 cells expressed elevated STAT3 at both the RNA and the protein level (Fig 3D). SiRNA-mediated knockdown of LMP1 and LMP2A resulted in slightly reduced expression of STAT3 (Fig 3E), demonstrating that EBV enhanced STAT3 levels via these factors. However, the impact of STAT3 on HLX in L-540 cells is primarily based on the subcellular localization of activated/phosphorylated nuclear/deacetylated STAT3 [6]. Therefore, we performed immunofluorescence analysis of phospho-STAT3 in EBV-positive/negative DOHH-2 cells (Fig 3F). Consistently, the nuclear levels of phospho-STAT3 were significantly higher in EBV-positive DOHH-2 cells. Moreover, we treated DOHH-2 cells with STAT3-inhibitors AG490 and TSA which reduce STAT3-phosphorylation and inhibit deacetylation-mediated nuclear import, respectively [6]. Both treatments resulted in decreased expression levels of HLX in L-540 and EBV-positive DOHH-2 cells (Fig 3G), supporting that activated/phosphorylated and nuclear/deacetylated STAT3 activates HLX expression in DLBCL cell line DOHH-2 as well.

STAT3 has been found to activate PRDM1 in the course of plasma cell differentiation [40]. Accordingly, treatment of EBV-positive DOHH-2 cells with STAT3-inhibitors AG490 and TSA resulted in decreased expression of PRDM1 as well (Fig 3H). Furthermore, siRNA-mediated knockdown of LMP1 (but not of LMP2A) led to downregulation of PRDM1 (Fig 3H). Thus, EBV-mediated activation of STAT3 performed increased expression of both, HLX and PRDM1.

### HLX regulates B-cell differentiation and apoptosis

**Fig 4:**
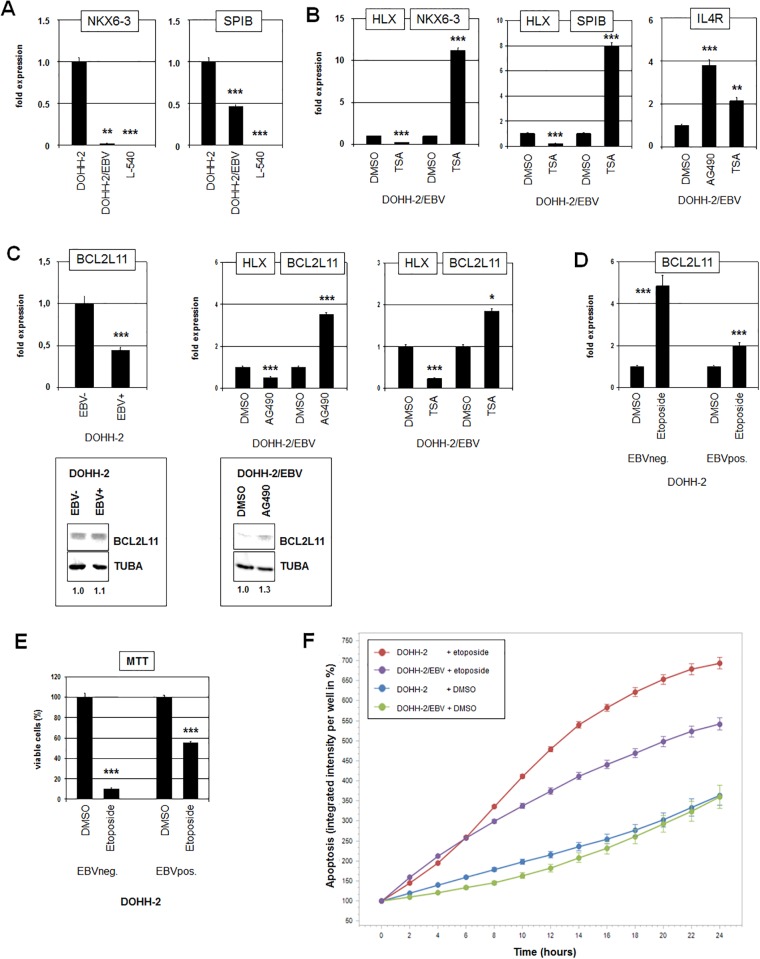
HLX suppresses differentiation and apoptosis. (A) RQ-PCR analysis of NKX6-3 (left) and SPIB (right) performed in EBV-negative and EBV-positive DOHH-2 cells in addition to L-540 cells. (B) RQ-PCR analysis of EBV-positive DOHH-2 cells treated with TSA for HLX and NKX6-3 (left), for HLX and SPIB (middle), and for IL4R (right). (C) RQ-PCR analysis of EBV-negative and EBV-positive DOHH-2 cells for BCL2L11 (left), and of EBV-positive DOHH-2 cells treated with AG490 (middle) or with TSA (right) for HLX and BCL2L11. Western blot analysis of BCL2L11 (below) was performed in EBV-negative and EBV-positive DOHH-2 cells (left) and in EBV-positive DOHH-2 cells after treatment with AG490 (right). Tubulin alpha (TUBA) served as loading control, densitometric calculations are indicated below. (D) RQ-PCR analysis for BCL2L11 of EBV-negative and EBV-positive DOHH-2 cells treated with etoposide for 16 h. (E) MTT-assay of EBV-negative and EBV-positive DOHH-2 cells treated with etoposide for 16 h shows significantly higher levels of viable cells in EBV-positive DOHH-2 cells. (F) Live-cell imaging analysis of EBV-negative and EBV-positive DOHH-2 cells treated with etoposide for 24 h. Apoptotic cells were quantified using the Caspase-3/7 Green Apoptosis Assay.

NKX6-3 belongs to the B-cell specific NKL-code and is the only code-member expressed in differentiated plasma cells while HHEX is solely expressed in memory B-cells [4]. Consistent with the suppressive impact of HLX on NKX6-3 the expression level of NKX6-3 was respectively downregulated and absent in EBV-positive DOHH-2 cells and in L-540 cells (Fig 4A). In addition to NKX6-3, HLX also regulates the B-cell specific differentiation factors SPIB and IL4R in these cells [4,41]. Accordingly, their expression levels were downregulated in EBV-positive DOHH-2 cells as well (Figs 2B and 4A). Therefore, to further examine the EBV-mediated impact on HLX-regulated B-cell development we analyzed EBV-positive DOHH-2 cells after treatment with STAT3-inhibitors AG490 and TSA (Fig 4B). These procedures resulted in reduced expression levels of HLX and concomitantly elevated levels of NKX6-3, SPIB and IL4R. Thus, EBV-mediated activation of HLX inhibits basic differentiation factors which normally regulate the development of plasma cells [4,42,43].

Furthermore, HLX represses in L-540 cells the expression of the pro-apoptotic factor BCL2L11/BIM [6]. Consistently, RQ-PCR analysis demonstrated significantly lower levels of BCL2L11 transcripts in EBV-positive as compared to EBV-negative DOHH-2 subclones (Fig 4C). Moreover, treatment of EBV-positve DOHH-2 cells with AG490 or TSA resulted in elevated expression levels of this gene at both the RNA and protein level (Fig 4C). Etoposide is a drug which induces BCL2L11 expression and subsequently apoptosis in malignant B-cells [44,45]. Accordingly, treatment of DOHH-2 cells with etoposide raised the expression levels of BCL2L11 (Fig 4D). Of note, this increase was significantly higher in EBV-negative DOHH-2 cells. To examine the apoptotic impact of etoposide we performed an MTT-assay after 12 hours of treatment (Fig 4E). These results indicated that the level of viable cells was significantly higher in EBV-positive than in EBV-negative DOHH-2 cells. In addition, we quantified the induction of apoptosis by etoposide via live-cell imaging in a time-course experiment for 24 h using a fluorescence-based caspase-assay (Fig 4F). The data showed that the percentage of apoptotic cells increased significantly stronger in EBV-negative than in EBV-positive DOHH-2 cells. Thus, EBV-mediated an anti-apoptotic effect in etoposide-treated DLBCL cell line DOHH-2 probably via activation of HLX and subsequent inhibition of BCL2L11.

## Discussion

**Fig 5:**
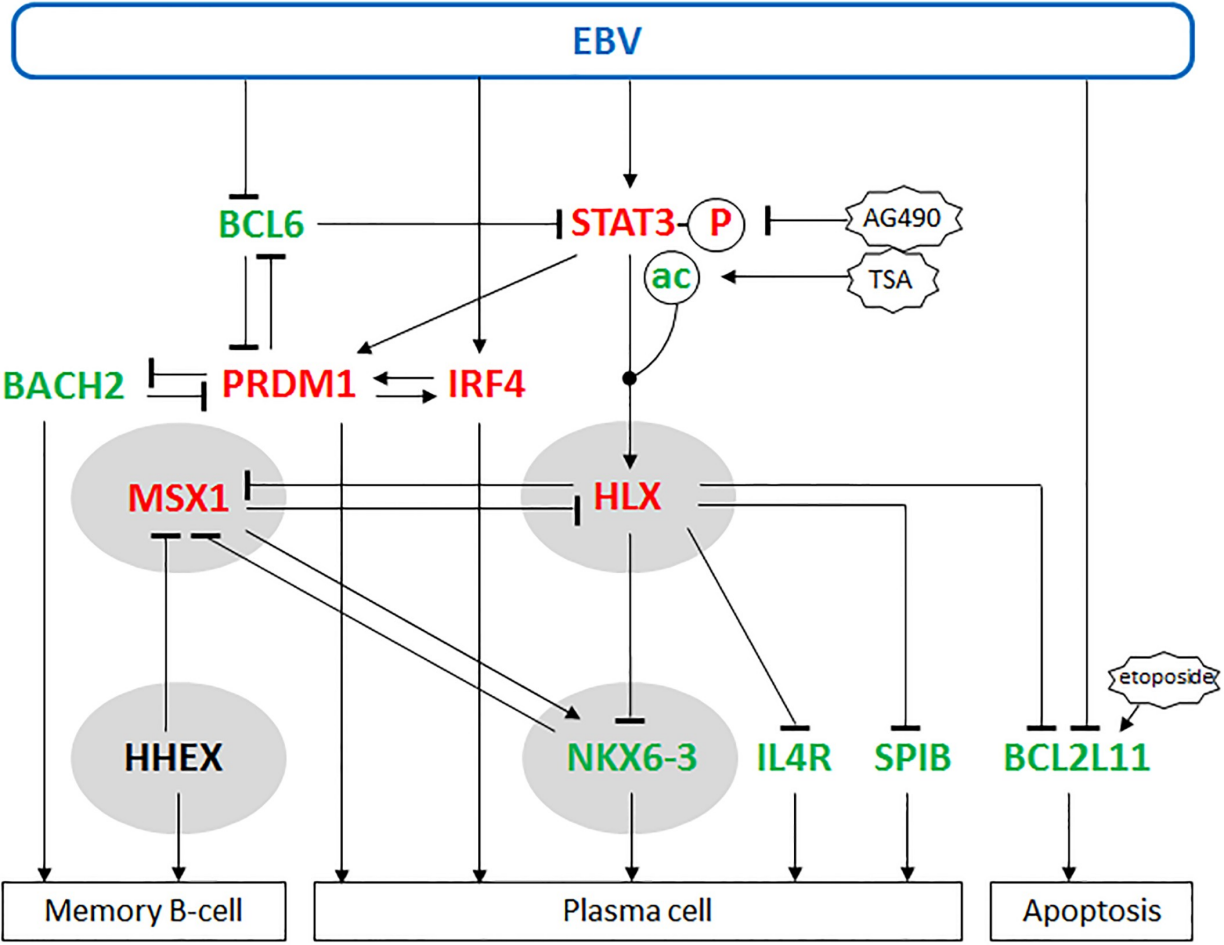
Summary of our study. This diagram combines the results of our study with data from the literature as indicated in the text. EBV activates IRF4 and STAT3 which in turn activates expression of NKL homeobox gene HLX. HLX is located in a central position of this network. Downstream targets of HLX are NKL homeobox genes MSX1 and NKX6-3, plasma cell factors IL4R and SPIB, and pro-apoptotic factor BCL2L11. Our data indicate that HLX inhibits plasma cell differentiation via NKX6-3, IL4R and SPIB and apoptosis via BCL2L11. Developmental regulators BCL6, BACH2, PRDM1 and IRF4 regulate each other and are targeted directly or indirectly by EBV. Upregulated genes/modifications in EBV-positive DOHH-2 cells are indicated in red, downregulated genes/modifications in green and genes showing no alterations in expression levels in black. NKL homeobox genes are boxed in grey.

A summary of the results of this study in combination with data from the literature is shown in Fig 5. We have established a cell line model to analyze the role of EBV in malignant B-cells. Isolated subclones of EBV-positive and EBV-negative DOHH-2 cells were used to investigate the impact of this virus on the B-cell specific NKL-code members in DLBCL. We showed that EBV-encoded LMP1 and LMP2A activated the expression of NKL homeobox gene HLX. This impact was mediated by activated/phosphorylated and nuclear/deacetylated STAT3. Raised levels of HLX inhibited the expression of the developmental regulators NKX6-3, IL4R and SPIB, and of the pro-apoptotic factor BCL2L11. The NKL-code member HHEX is involved in the differentiation of memory B-cells and was not altered by EBV. In contrast, NKX6-3 in addition to IL4R and SPIB mediate plasma cell differentiation; these three factors were repressed by EBV via HLX. Therefore, according to these analyzed genes EBV-infected B-cells shifted their developmental potential towards memory B-cells and were less sensitive for etoposide-induced apoptosis. However, reported impacts of EBV include suppression of BCL6 and activation of IRF4. These factors together with STAT3 are part of a deregulated network also containing BACH2 and PRDM1 [46]. BACH2 mediates differentiation of memory B-cells while PRDM1 and IRF4 support plasma cell differentiation including activation of CD138 [47,48]. Their altered expression levels in EBV-positive DOHH-2 cells correspond to the status of plasma cells contrasting the outcome of HLX activation. Together, our results revealed particular effects of EBV-mediated HLX activation in B-cell differentiation and survival which may play an important role in EBV-positive DLBCL. HLX represents an additional mediator but no master-regulator of these processes. Accordingly, EBV infection of DOHH-2 cells deregulated several developmental genes which, however, show no consistent picture of the differentiation status.

The EBV-encoded factors LMP1 and LMP2A activate STAT3 in B-cells as indicated here and reported previously [37–39]. The transcriptional activity of STAT3 is regulated via phosphorylation and acetylation. The latter modification supports nuclear export as shown in DLBCL and HL cells [6,49]. Consistently, our data obtained in DLBCL cell line DOHH-2 indicated that phosphorylated and deacetylated STAT3 is located in the nucleus and activates the expression of its target genes HLX and PRDM1. Accordingly, expression profiling data of EBV-positive and EBV-negative DLBCL and HL patients showed higher transcript levels of HLX in EBV-positive patients of both lymphoma entities (S2 Fig). However, the differences of these patient data were not statistically significant. One explanation for this weak association might be that in addition to EBV other oncogenic factors operating in lymphoma cells influence STAT3-signaling and HLX expression as well.

DLBCL has been divided into GC- and ABC-subtypes [50]. EBV is associated more frequently with the non-GC or ABC-type of DLBCL which demonstrates elevated STAT3 and NFkB activation [51,52]. NFkB is an activator of EBV-encoded LMP1 which in turn activates STAT3 [37–39,53]. Consistently, NFkB-overexpression is associated with ABC-DLBCL and EBV-positive DLBCL cases [54]. On the other hand, BCL6 operates as transcriptional repressor and has been shown to suppress STAT3 and PRDM1 expression [53, 55]. Accordingly, EBV-positive DOHH-2 cells expressed decreased levels of BCL6 and elevated levels of PRDM1 and STAT3. Recently, a particular mutation in the MYD88 gene has been identified to mediate STAT3 activation in ABC-DLBCL which gives a rational explanation for this relationship [56]. Thus, EBV infection is one factor among others in DLBCL which influences the activity of STAT3-signalling.

Memory B-cells and plasma cells represent final stages in the course of B-cell development [3]. Plasma cell differentiation is mediated via IL4-signaling and requires the transcription factors BACH2 and SPIB [42,43,57]. The activity of NKL-code member NKX6-3 is associated with the plasma cell status and may be, therefore, functionally implicated in according differentiation processes as well [4]. The infection of B-cells with EBV inhibits their differentiation into plasma cells [30]. These data are in accordance with our results, showing that BACH2 is downregulated in EBV-positive DOHH-2 cells and that HLX operates as an inhibitor of IL4R, SPIB and NKX6-3. In addition to B-cells, normal expression of NKX6-3 has been described in the gastric mucosa and in neural crest cells [58,59]. Moreover, a crosstalk between NKX6-3 and MSX1 takes place in both, B-cell differentiation and neural crest development [4,59]. In gastric cancer cells NKX6-3 acts as a tumor suppressor and inhibits NFkB [60]. This function may also play a role in malignant B-cells, suppressing ABC-DLBCL which exhibits enhanced NFkB-activity [61]. Thus, NKX6-3 may perform tumor suppressor activity in ABC-DLBCL as well.

BCL2L11/BIM acts as a central apoptotic factor in normal and malignant B-cells [44,45]. The apoptotic activity of BCL2L11 is frequently suppressed in B-cell lymphomas by overexpression of interacting factors like BCL2. A common mechanism of BCL2 activation represents the chromosomal translocation t(14;18)(q32;q21) which juxtaposes the loci of IGH and BCL2. This rearrangement is frequently associated with another translocation, t(8;14)(q24;q32), targeting MYC [62,63]. Downregulation of BCL2L11 by genomic deletion, DNA-methylation or via overexpressed micro-RNAs targeting BCL2L11 represent alternative or additional mechanisms in B-cell lymphomas to escape apoptosis [64–66]. Our data indicate that phosphorylated and deacetylated STAT3 activates HLX which in turn inhibits BCL2L11 expression, representing a novel mechanism of BCL2L11 suppression in DLBCL. Consistently, histone deacetylase inhibitors induce apoptosis in EBV-positive B-cells and in ABC-type DLBCL cells [49,67], supporting the clinical relevance of the findings in this study. Inhibition of BCL2L11 transcription by EBV is also performed via epigenetic alterations including methylation of DNA and histones [65,68]. These impacts are mediated by the viral factors EBNA3A and EBNA3AC. Transcripts of EBNA3A/C were also detected in EBV-positive DOHH-2 cells which indicates that both, HLX and these EBV-factors suppress BCL2L11 expression, thus, representing alternative and synergistic modes of aberrant regulation of cell survival.

Together, our data reveal a novel player underlying the oncogenic impact of EBV in differentiation and survival of B-cells. We show that NKL homeobox gene HLX occupies a central position in basic EBV-mediated effects in infected malignant B-cells. In addition, the EBV-positive/negative subclones of DOHH-2 show several deregulated developmental genes involved in B-cell differentiation. Therefore, these cell lines may serve as suitable model to investigate the role of EBV in particular aspects of B-cell malignancies.

## Supporting information

S1 Fig Authentication of DOHH-2 subclones. STR profiling data are shown for DOHH-2 reference cell line (above), and for EBV-positive and EBV-negative DOHH-2 subclones (below), demonstrating their identity.
(TIF)

S2 Fig HLX expression in EBV-infected HL and DLBCL patients. Public expression profiling data of EBV-positive and EBV-negative (A) HL patients (GSE3996) and (B) DLBCL patients (GSE17372) show elevated HLX expression in EBV-positive patients as compared to EBV-negative controls in both entities. However, the p-values (obtained by using the Mann-Whitney-U Test) indicated absence of statistical significance.
(TIF)

S1 Table Comparative expression profiling data showing the top-1000 overexpressed genes in EBV-negative DOHH-2 cells. (XLS)

S2 Table Comparative expression profiling data showing the top-1000 overexpressed genes in EBV-positive DOHH-2 cells. (XLS)
