# Supplementary figures and images for: Epstein-Barr virus (EBV) activates NKL homeobox gene HLX in DLBCL
Source: PLoS One. 2019 May 29;14(5):e0216898. doi: 10.1371/journal.pone.0216898 (PMC6541347; doi:10.1371/journal.pone.0216898)

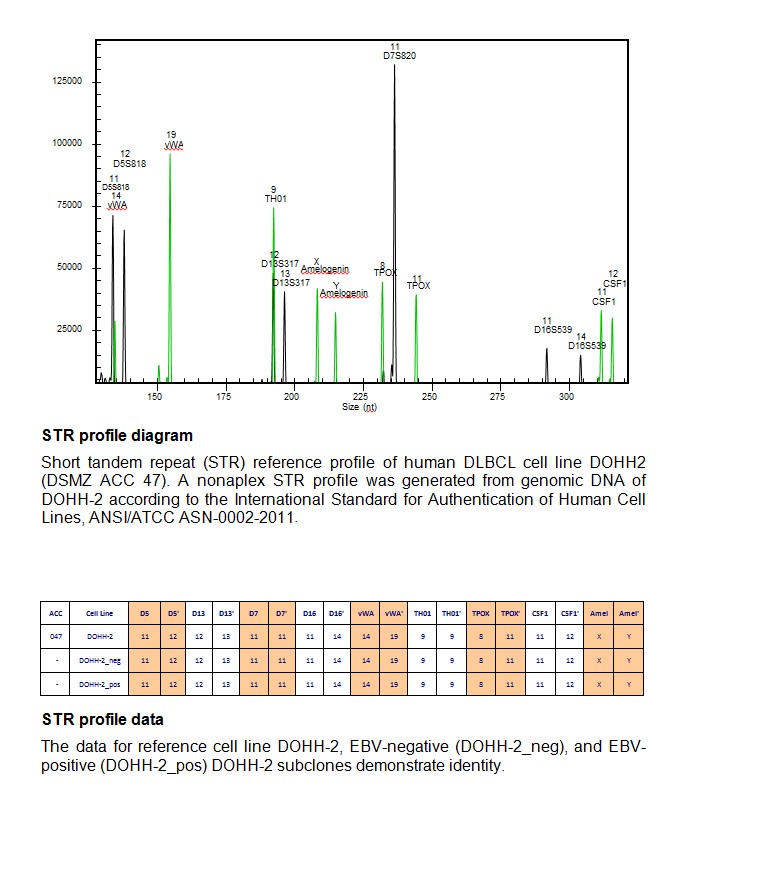

Supplement: S1 Fig — STR profiling data are shown for DOHH-2 reference cell line (above), and for EBV-positive and EBV-negative DOHH-2 subclones (below), demonstrating their identity. (TIF) [file pone.0216898.s001.tif]

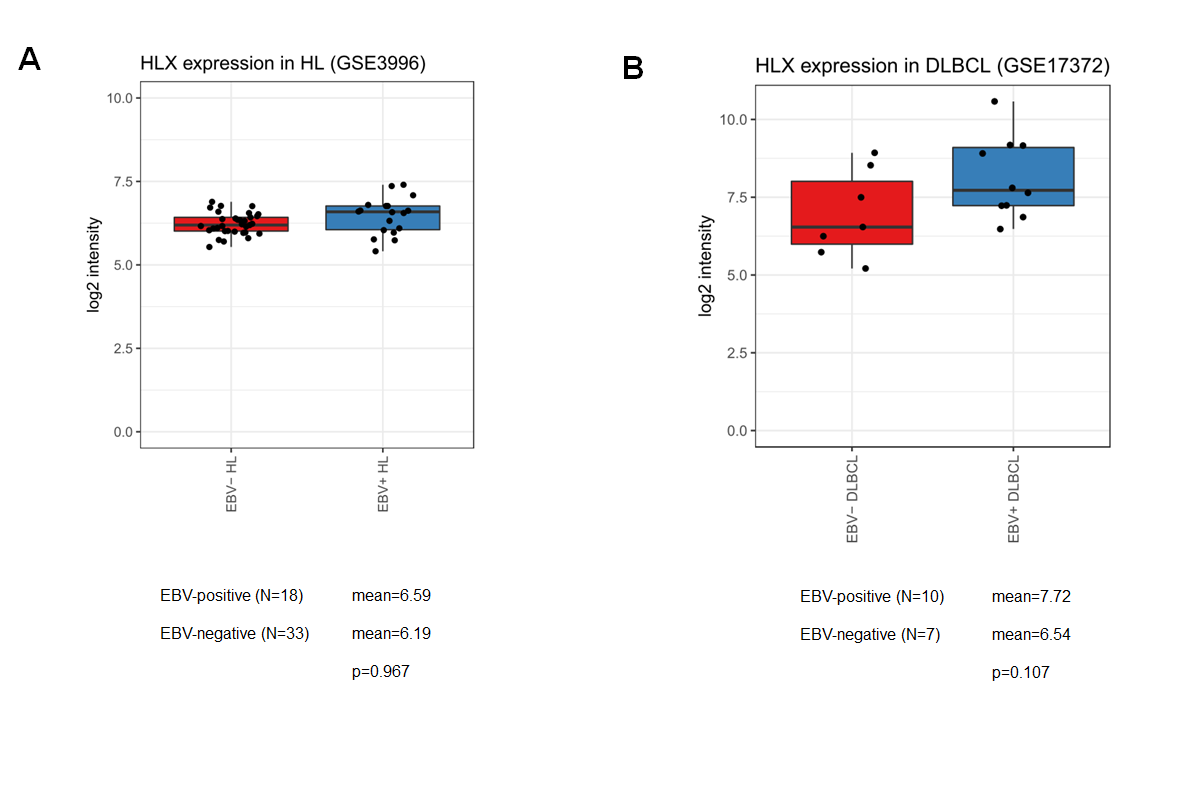

Supplement: S2 Fig — Public expression profiling data of EBV-positive and EBV-negative (A) HL patients (GSE3996) and (B) DLBCL patients (GSE17372) show elevated HLX expression in EBV-positive patients as compared to EBV-negative controls in both entities. However, the p-values (obtained by using the Mann-Whitney-U Test) indicated absence of statistical significance. (TIF) [file pone.0216898.s002.tif]
